# Risk Factors Based on Analysis of Injury Mechanism and Protective Equipment for Ice Hockey Amateur Players

**DOI:** 10.3390/ijerph19074232

**Published:** 2022-04-01

**Authors:** Heejae Jin, Hyojeong Lee

**Affiliations:** Department of Fashion Design and Merchandising, Kongju National University, Gongju 32588, Korea; heejaejin@gmail.com

**Keywords:** ice hockey, amateur, injury, risk factor, protective equipment

## Abstract

Considering the increasing popularity of ice hockey in South Korea, types of injuries and protective equipment for amateur club members need to be further studied. The purpose of the study is to investigate various injuries and protective equipment used by amateur players and to analyze different risk factors by collecting information on areas of injury and their mechanisms. The online survey for 102 participants was designed to address the general information of participants, types of injuries, information about ice hockey equipment, and open-ended questions about injuries and equipment. We conducted in-depth face-to-face interviews with five players about their experiences with injury and opinions about the protective equipment. In total, 60% of the survey participants had experienced injuries, including to the knee (22.6%), shoulder (21.6%), ankle (20.6%), and wrist (14.7%), in order of frequency. Types of injury included sprain (33.3%), contusion (31.4%), fracture (17.7%), abrasion (10.8%), and concussion (0.9%). Injury mechanisms included instances in which those with less proficiency in skating would be injured initially from player-to-player contact, and from landing on the ice or crashing into the fence afterward. We acknowledged how important wearing correctly sized equipment is for protection, and we highlighted the need to develop protective gear that accommodates Asian body measurements.

## 1. Introduction

Ice hockey is a high intensity sport involving high speed and frequent physical contact. While ice hockey is most popular in Canada, the U.S., and northern Europe, it is currently enjoyed and played by most nations around the world [1]. In Korea, the sport is also recently being played by an increasing number of club members whose age varies widely between 20–70 years. The amateur club leagues are also highly competitive, further demonstrating the growth in popularity of the sport [2].

Ice hockey is a combination of high speed with the use of sticks, a fast-moving puck, and physical contact among players, which predisposes athletes to the risk of serious injury. It is also a sensitive sport that can result in risk of injury from even the slightest inattention [3,4]. In order to prevent the potentially high risk of injury, ice hockey players wear protective gear over the entire body. Thus, wearing impact protective clothing and equipment is essential while playing ice hockey. The protective equipment used in ice hockey includes helmets, mouth guards, shoulder pads, elbow pads, padded gloves, padded pants, and shin guards. Even with such impact protective equipment over the whole body, the risk of injury is still considerably high. Head injuries are also dangerous, which explains why the majority of research related to injury and protective wear are based on cerebral concussions and dental injuries [5,6,7,8,9,10,11]. On the other hand, research on injury of other body parts demonstrated results as follows. The distribution of typical ice hockey injuries in the American NHL and Canadian and European leagues showed knee injuries at 40%, shoulder injuries at 20%, groin injuries at 15%, and back injuries at 10% [12]. An analysis of traumatic injuries and overuse injuries of high-level junior ice hockey players showed that injuries of the hip and lower limbs such as the thigh and knee were 32% prevalent, upper limbs such as the shoulder, wrist, and hand were 31%, and the rest involved the head, neck, and the trunk [13,14]. The shoulder was also one of the most frequently injured areas for Korean high school players, and the knee was the most common cause of hospital visits [15]. Thus, typical ice hockey injuries frequently include the knee, shoulder, wrist, and other various areas. There are some studies analyzing injury mechanisms and patterns in order to prevent injuries. Collision with an opponent, body checking, stick and puck contact, and falling were frequent mechanisms for ice hockey injuries [3]. As aggressive act was the cause for primary injury in young boys; injuries were reduced by changing mandatory rules and educational and cognitive behavioral interventions to decrease aggression [16,17,18]. In addition, a study about the association of gender and injuries showed that male hockey players have higher rate of injury than women due to accepting body checking for the male players, although the predominant mechanism of injury was via direct contact for both male and female players [19]. Thus, it was shown that the area, mechanism, and prevention of injury depends on the characteristics of subjects of each study. In this regards, Asian amateur ice hockey club members can be seen as a unique characteristic for analyzing injuries. However, there is a lack of recent research on this important topic.

While the impact protective equipment in sports is critical for protection of the body, the effect of exercise and the performance of a player can be reduced and risk of injury may increase if not worn properly. As expected, there have been occasional reports of how the protective equipment is inconvenient to use, how the fit is incompatible, and how it hinders movement and adds to discomfort due to thermal stress [20,21,22,23] Between various ice hockey protective equipment, there has been relatively more research on the helmet and mouthguard, with their fit and significance highly emphasized [24,25,26,27]. However, there has been a dearth of research on ice hockey protective equipment concerning the entire body. Nevertheless, for amateur ice hockey players with less experience in skating or participating in games, the effect of overall protective equipment needs to be considered, as the protection that the equipment provides can have a far greater significance. Considering the increasing popularity of ice hockey between non-trained children and adults, types of injuries and protective equipment for less skilled club members need to be further studied.

Thus, our purpose is to study various injuries and protective equipment used by amateur players in South Korea who participate in ice hockey more as a hobby for enjoyment. Additionally, we seek to analyze different risk factors by collecting information on areas of injury and their mechanisms along with use of protective equipment based on the years played. Furthermore, the data collected in our study can contribute to further pursuits to prevent injury for the amateur players.

## 2. Method

### 2.1. Survey for Data Collection

**Figure 1:**
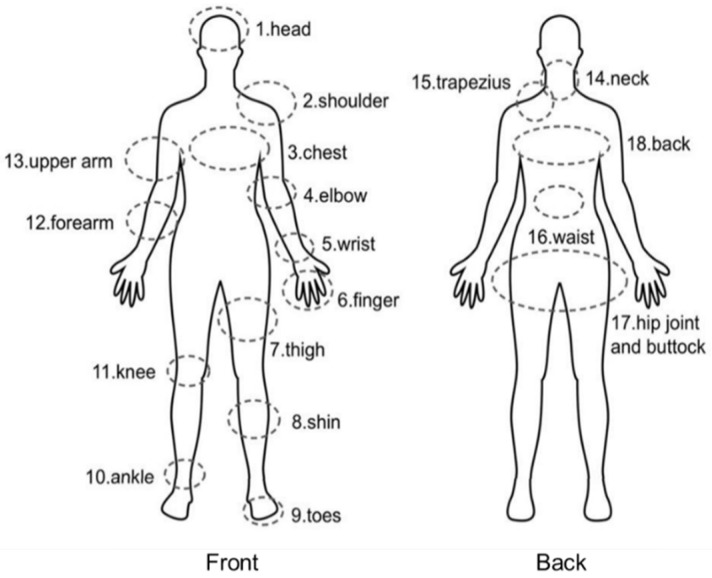
Area of injury.

Upon IRB approval (2022-008) from the researchers’ university, ice hockey amateur players were recruited. We selected participants through a random sampling process and asked them to participate. Afterwards, they completed the online survey using the URL link (NAVER Formβ online survey service, NAVER Corp., Seongnam-si, Korea). The participants recruited were enrolled in ice hockey clubs in Seoul and Daejeon, South Korea. The number of participants was 102, and their age range was 20–60 years. The questionnaire based on the literature review consisted of four sections [8,13,15,16]. Section 1 was designed to address the general information of participants. Section 2 assessed the data of injuries. In this section, participants were allowed to choose multiple areas of injury on the given body illustration (Figure 1). Section 3 addressed the information about the replacement of equipment for ice hockey. Participants responded with what factors they place priority in when they replace each equipment, from first to third. In addition, the principal equipment that participants choose to replace first in regard to protection was addressed in this section. Section 4 included open-ended questions about the injuries and equipment.

### 2.2. Data Analysis

We used SPSS 24.0 (IBM software, New York, NY, USA) for statistical analysis of frequency analysis and multiple response analysis. In order to analyze the replacement of protective equipment, the rankings were calculated with weighted values for each ranking order. The ranking system was 1st, 2nd and 3rd, with a score of 5, 3, and 1 given, respectively. The total order score rankings were then made up from the sum of all the scores. A Chi squared test was implemented with the ice hockey players’ characteristics as independent variables and experience of injury as the dependent variable in order to assess their relationships. We analyzed participants’ statements in open-ended questions by using NVivo 12.0. Data was analyzed and coded with context analysis as the qualitative method [28]. Researchers repeatedly read the collected data and directly categorized them by specific topics. In addition, coding created with implied categorized meaning was achieved [29]. In this step, we discussed for to ensure agreement of our opinion, and the code was compared and analyzed. The frequency of topic words were identified through the content analysis by the coding. For the validity of the process and result of the study, peer review was used. NVivo 12.0 can clearly show the data process of the study in the qualitative research, and help find the quantitative value.

### 2.3. In-Depth Interview

We conducted in-depth face-to-face interviews as there was a limitation to sufficiently draw out the opinion of amateur players using only self-reported surveys. Information about injury experiences and ice hockey equipment were hardly collected through surveys, thus being collected through interviews. Using convenience sampling, which collects data from a conveniently accessible population, we recruited 5 players from an amateur ice hockey club (Ice Unicorns in Daejeon) for an in-depth interview. We requested permission to conduct the in-depth interview and provided details about the study to individuals who agreed for the interview. The researcher audio recorded each interview with permission, as well. The study team organized interview questions into two sections based on the results from statistical analysis. In the first section, questions included: Do you have any experience of injuries? If so, can you explain the situation? What is your effort to prevent injuries? Questions in the second section included: How does the size of the protective equipment fit you as a Korean? What is your thought about the safety of the protective equipment? Characteristics of participants are shown in Table 1. The result of the in-depth interview was analyzed by coding using NVivo 12.0. The procedure was similarly applied with the above mentioned analysis in the open-ended questions. In particular, coding was separately implemented according to each of the questions and subtopics, including sentences or phrases that were grouped into subcategories of related codes. Then, the subcategory was grouped into the category of related subcategory, which was then grouped together into the emergent theme through the inductive method.

## 3. Results

### 3.1. Characteristics of Survey Participants

Characteristics of participants are listed in Table 2. A total of 102 participants replied, with 80 male participants (78.5%) and 22 female participants (21.6%). Participants had various occupations including 25 working in the professional field (24.5%), 23 office workers (22.6%), 17 self-employed (16.7%), 11 college students (10.8%), and others involved in education, service, and administration.

The age range of participants was between 20–60 years, including 55 individuals aged 40 years (53.9%) and 24 aged 30 years (23.5%). Of these participants, 35.3% indicated playing ice hockey for more than 10 years, 34.2% for 1 to 5 years, 17.7% for 5 to 10 years, and 12.8% reported playing ice hockey for less than 1 year. Additionally, 50% of participants played less than 4 times per month, 32.3% played 5–9 times per month, and 17.8% played more than 10 times per month.

### 3.2. Experience of Injury

Based on the analysis of injury information (Table 3), this study showed that 60 (58.9%) out of 102 participants have been injured during play at the ice hockey club. For the area of injury, the distribution showed knee injuries to be 22.5%, shoulder 21.6%, ankle 20.6%, wrist 14.7%, waist 8.8%, chest and finger each 7.8%, as well thigh, neck, hip joint, and buttock each 4.9%. Meanwhile, there were rarely injuries involving the head, elbow, or toes. The majority of the type of injury was sprain 33.33% and contusion 31.3%, followed by fracture 17.6% and abrasion 10.7%. Although only one case of a concussion emerged within the study, it is frequently shown that it is common amongst professional ice hockey players. In order to ascertain the relationship between the experience of injury and the amateur hockey players’ characteristics, a Chi squared test was conducted (Table 4). Out of the participants who have played for more than 5 years, 75.5% experienced injury, while 60.4% (*p* = 0.001)) of participants who have played for less than 5 years had no experience of injury to date. In addition, 65% of male participants had experienced injury, whereas 68.2% (*p* = 0.000) of female participants had no experience of injury.

### 3.3. Replacement of Protective Equipment

To better understand the replacement of protective equipment, two categories were analyzed: priority factors to consider for replacement and principal equipment replaced first in regard to protection (Table 5). In the priority factors considered for the replacement of equipment, we found that protective function was a major consideration factor, followed by price, weight, size, brand, and design. Additionally, in the aspect of the principal equipment selected for replacement, participants indicated that they choose to replace the shin guard first for protection, followed by pants, shoulder pads, jock pants, elbow pads, and neck guard. Based on the result of the injury distribution which revealed that knee injury occurred most frequently, it was shown that the participants used their experiences with injury when choosing to replace the shin guard first for protection.

### 3.4. Response of Open-Ended Questions

The respondents were asked to freely describe their opinions at the end of the survey. Thirty-eight out of one hundred two participants offered their impression. Table 6 represents the analytical results using ‘open coding method’. Size, management, wearing, the protective level of equipment, and price for the ice hockey protective equipment were classified. The protective level of equipment has a positive correlation with the price, and respondents believe that more expensive equipment is more comfortable (n = 12, 31.6%). In addition, they expressed discomfort due to the heavy weight and how the elbow pad or the shin guard would slip down (n = 13, 34.2%). For the size, participants felt that the ice hockey equipment being mostly from overseas was too large for the Korean measurements. Along with the fact that the equipment was too big on the body, the inability to try the equipment on was also noted (n = 10, 26.3%).

### 3.5. In-Depth Interview

**Figure 2:**
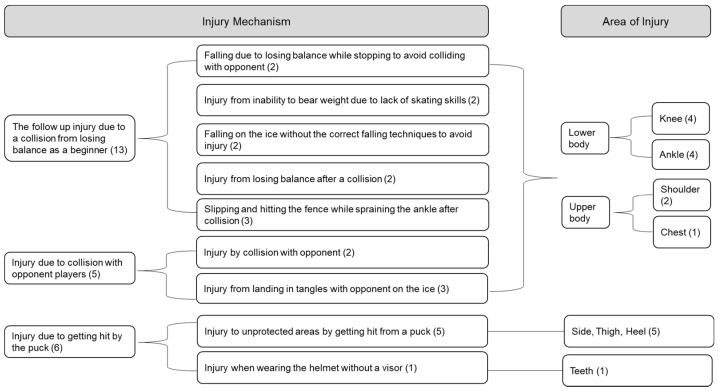
Injury mechanism and area of injuries (coding number in parenthesis).

The response results from in-depth interview were coded according to question, and extracted topics were categorized and analyzed. Injury mechanism and the area of injury were represented with the diagram shown in Figure 2. Injury mechanism was analyzed with three categories, which were the follow up injury due to a collision with opponent players from a lack of skating skills by losing balance, direct collision with an opponent player, and being hit with the puck. All the interviewees have had experiences with wounds from pucks hitting areas without protection, such as the teeth, inner and outer thigh, feet, flank, etc., as well as knee, ankle, shoulder, and chest injuries after collision. Particularly, P1 and P2 also mentioned experiencing shoulder trauma. Such injuries were usually due to slipping and falling on the ice or crashing onto the fence after colliding with an opponent. Bumping with an opponent can also directly lead to ankle injury after entangling and falling onto each other.

**Figure 3:**
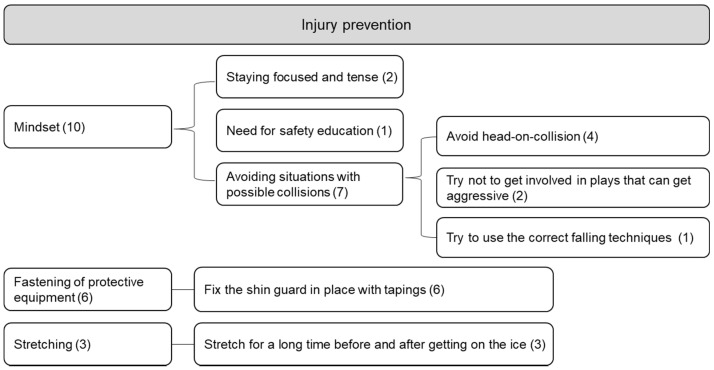
Prevent injuries (coding number in parenthesis).

For responses of preventing injuries (Figure 3), mindset, stretching, and fastening of protective equipment were classified as three categories. Interviewees emphasized a mindset involved in staying focused and tense, the necessity for safety education, and avoiding collision circumstance. In order to avoid collision, P3 and P4, with 10 years of ice hockey experience, described how they intentionally fall on areas that are well-protected in order to avoid head-on-collisions. They pointed out the decrease in frequency of injuries compared to when they were beginners. Thus, the ability to react proficiently to risk of injury varies based on one’s experience and skills. Additionally, they highlighted their habits of adding taping and banding in order to secure on the shin guards. While the equipment usually allows for safe games, knee injuries can result from shin and elbow guards sliding down and around. In addition, based on such experiences, participants explained how they made sure to stretch and relax the body enough before playing.

**Figure 4:**
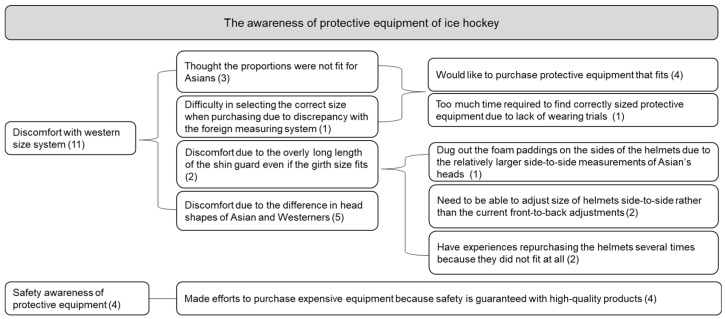
The awareness of protective equipment for ice hockey (coding number in parenthesis).

Figure 4 categorized the opinion of protective equipment into two big groups: discomfort felt by Asians due to the size system made for the Western population, and the safety awareness of protective equipment. Interviewees required fitted protective equipment because they were not familiar with Western size systems and are structurally out of proportion. Moreover, there were notable discomforts due to the difference in head shapes between Asians and Westerners. Amongst the Koreans, there were many complaints about the helmet when it came to opinions regarding size of the equipment. Every interviewee responded that it is difficult to find a properly sized helmet considering how the head is shaped differently between Asians and the Western population. The helmets which are adjustable for front-to-back size differences were a better fit for Westerners and failed to accommodate for side-to-side differences of the heads of Asians. Even helmets that could be adjusted side-to-side tended to be much smaller than needed. Likewise, P1, P2, and P4 replied that the shin guards designed according to the Western populations were much too long for their legs. In regards to the safety of the equipment, every participant thought that they were very safe, but emphasized how important it is to select the correctly sized equipment for the body.

## 4. Discussion

Ice hockey is a fast sport on a slippery surface with a high risk of injury due to collisions. Even professional ice hockey players with substantial experience and who display great performance can easily be injured. Our study showed that 60% of the surveyed participants had experienced injuries, including to the knee, shoulder, ankle, and wrist, in order of frequency. Such results were similar to those from North American and European professional league players which showed that the knee (40%) was injured the most, followed by the shoulder (20%), groin (15%), and back (10%) [1]. In addition, amateur ice hockey players in Switzerland showed the most injured area as the knee (17.9%), foot (14.3%), head (12.5%), and shoulder (10.7%) [30]. Listola et al. [13] reported that shoulder (20.3%), groin/hip region (20.3%), thigh (13%), and knee (5%) injuries occurred most commonly in elite junior ice hockey players, and that lower limb injuries occurred over fifty percent of the time. For Korean high school players, the maximum hospital visits per year by injury sites were knee (50 visits), foot and ankle (32 visits), and back (30 visits) [15]. Knee injury was the most frequent for ice hockey players in all ages in Sweden, and was the highest incidence of permanent medical impairment in female ice hockey players. Other research also showed the lower limbs as the area seeing the most injuries during ice hockey, especially the knee. Although it appeared that professional players had the highest incidence of injury to the head and neck due to aggressive body checking [31], head injury did not seem prominent in this study. This result deems that amateur players play less aggressively during the game. Furthermore, male club members received injuries more frequently. The male players had higher aggressiveness and hostile acts, and these qualities were shown to be the primary etiological factor for injury in ice hockey [32]. The precise mechanisms of injuries were made clear through the in-depth interviews. Excluding the coincidental hits from an airborne puck, collisions with other players led to most of the injuries. There were instances in which those with less proficiency in skating would be injured initially from player-to-player contact, but they would also be wounded from landing on the ice or crashing into the fence afterwards. The most frequent mechanisms for ice hockey injuries were shown to be collision with an opponent, body checking, stick and puck contact, and falling [3]. While Kuzuhara et al. [33] claimed that contact with the hockey stick was the most frequent mechanism, as amateur players do not engage in games that are as aggressive as those played by professional players, most of the injuries seemed to be due to the unintentional collisions with other opponents. Players experienced with such situations would focus and maintain a defensive attitude to avoid such contact. It is essential for amateurs to build their basic skills through continuous skating practice, as they lack the training for matches and skating. As described in the in-depth interviews, unlike the beginners, advanced players avoid injuries by intentionally falling on well-protected areas after collisions; amateur players should be given training according to their level of experience and skill. A decrease in aggressive actions reported to help significantly reduce injury rate, which were the mandatory rules involving the intended decrease of aggression, educational interventions, and cognitive behavioral modification [18,34]. As injuries were highly correlated with psychological factors, environmental factors, as well as physical factors for the collision [15], it is recognized that amateur players need safety education with the mindset of intervention in order to effectively prevent injury. Thus, upon joining the club, one would be able to avoid severe injuries with the education to prevent injury and a defensive mindset during the games.

The factors that survey participants placed priority in when using the equipment were the level of protection provided, cost, and size, in order of importance. The level of protection provided was especially emphasized in shin guards, followed by pants, then shoulder pads. This correlates significantly with the previously determined fact that the knee and shoulders were the most frequent area of injury. The details were likewise made clear through the in-depth interviews. In order to avoid injury, there were efforts to stretch sufficiently before the games and skate defensively, as well as take extra steps to wear the protective equipment. Especially when putting on the shin guard that is supposed to protect from the knee and the anterior portion of the shin to the ankles, participants had to add tapings and bandings to prevent the protective shell from rotating out of place during play. Several previous studies showed how wearing the protective equipment is uncomfortable and restricts the range of motion when playing [18,35]. In this respect, the amateurs experienced significant discomfort from the shin and elbow guards sliding down or moving around. Most of the shin and elbow guards up to the present use elastic bands and Velcro to secure them in place from the posterior side [3]. The fact that the protective equipment fails to stay in place calls for serious consideration of possible solutions regarding its technical design. Furthermore, while there was much discontent in respect to the size of equipment all from Western brands, especially concerning the shape and structure of helmets and the proportions of shin guards, participants were satisfied with the overall safety of the equipment. Thus, the need to develop protective gear that accommodates for Asian body measurements was highlighted. Boorady [36] claimed that poor fitting not only diminishes the protective function of the equipment but also exacerbates the problem of the pad shifting. Such problems were similar to those pointed out in this study.

## 5. Conclusions

We conducted a survey and an in-depth interview for amateur club members on ice hockey injuries and use of protective equipment. Qualitative and quantitative findings indicated that ice hockey amateur players had the most injuries in the knee, shoulder, and ankle, and how lack of experience and skill contributes significantly to the risk of injury. Club members were satisfied with the overall safety when wearing the equipment properly. In regard to the technical design, however, we addressed the problem with securing the shin and elbow guards in place, and with developing helmets that can accommodate for the Asian measurements.

By conducting research about the injuries and protective equipment used by Asian amateur ice hockey players, our results can contribute to providing a safer environment for the increasing number of ice hockey club members.

This study was limited to amateur ice hockey players located in Daejeon city in South Korea, which was not a large enough sample size to sufficiently generate for statistical inference. This study was also limited by recall bias with the retrospective data collection. However, it is meaningful that the result of this study is valuable for public health development, and has derived the needed further research with consideration to develop protective equipment design for the Asian body size.

## Figures and Tables

**Table 1:** Description of Participants of Interviewed.

	Sex	Years Played	Age Group	Number of Times Played Per Week
P1	Male	5 years	40s	3 times
P2	Male	4 years	40s	2 times
P3	Male	10 years	40s	3 times
P4	Female	10 years	30s	1 time
P5	Male	15 years	40s	5 times

**Table 2:** Characteristics of Survey Participants.

Category		N	(%)
Sex	Male	80	(78.4)
	Female	22	(21.6)
Occupation	College students	11	(10.8)
	Working in professional field	25	(24.5)
	Administration	6	(5.9)
	Office worker	23	(22.6)
	Self-employed	17	(16.7)
	Others	20	(19.6)
Age group	20s	15	(14.7)
	30s	24	(23.5)
	40s	55	(53.9)
	50s	6	(5.9)
	60s	2	(1.9)
years played	Less than 1 year	13	(12.8)
	1–5 years	35	(34.2)
	5–10 years	18	(17.7)
	More than 10 years	36	(35.3)
number of times played per month	Less than 4 times	51	(50.0)
	5–9 times	33	(32.4)
	More than 10 times	18	(17.6)

**Table 3:** Injury Information of Participants.

Category		N	(%)
Experience of injury	Yes	60	(58.8)
	No	42	(41.2)
Area of injury	Knee	23	(22.6)
	Shoulder	22	(21.6)
	Ankle	21	(20.6)
	Wrist	15	(14.7)
	Waist	9	(8.8)
	Chest	8	(7.8)
	Fingers	8	(7.8)
	Others (thigh, neck, hip joint, head, etc.)	23	(17.8)
	Total	129	(126.5)
Type of injury	Fracture	18	(17.7)
	Contusion	32	(31.4)
	Sprain	34	(33.3)
	Abrasion	11	(10.8)
	Concussion	1	(0.9)
	Others	9	(8.9)
	Total	105	(102.9)

**Table 4:** Injury Experience based on Years Played and Sex of Participants.

		Experienced Injury		Have No Experience of Injury		χ^2^	*p*
		N	(%)	N	(%)		
Year played	Less than 5 years	20	(39.6)	29	(60.4)	11.212	0.001
	More than 5 years	39	(75.5)	14	(26.4)		
Sex	Male	53	(65.0)	27	(33.8)	59.941	0.000
	Female	7	(31.8)	15	(68.2)		

**Table 5:** The Aspect of the Replacement of Protective Equipment.

		**Protective Function**	**Price**	**Weight**	**Size**	**Brand**	**Design**
Priority factors to consider for replacement	First	48	19	5	12	8	10
	Second	31	30	16	12	5	8
	Third	14	31	13	16	12	16
	Total order score ^a^	347	216	86	112	67	90
	Rankings	1	2	5	3	6	4
		**Jock** **Pants**	**Shin Guard**	**Elbow Pad**	**Shoulder Pad**	**Pants**	**Neck Guard**
Principal equipment chosen for replacement when considering protection	First	21	31	6	18	23	3
	Second	5	37	17	21	17	5
	Third	7	13	30	17	33	2
	Total order score ^a^	127	279	111	170	199	32
	Rankings	4	1	5	3	2	6

^a^ Total order score = 1st(n) × 5 + 2nd(n) × 3 + 3rd(n) × 1.

**Table 6:** The categories for the open-ended questions about ice hockey equipment (n = 38).

Category (n, %)	Subtopics	Frequency (n, %)
Size (n = 10, 26.3%)	Discomfort due to the different size system especially if worn without testing it on	(n = 9, 23.6%)
	For women, finding the right fitted size	(n = 1, 2.6%)
Management (n = 3, 7.8%)	Dissatisfaction due to inability of washing/cleaning equipment	(n = 3, 7.9%)
Wearing (n = 13, 34.2%)	Discomfort due to heavy weight	(n = 8, 21.0%)
	Discomfort from elbow pad or shin guards slipping	(n = 5, 13.1%)
Equipment of protective level and price (n = 12, 31.6%)	Increased protective ability of equipment means increased price.	(n = 6, 15.8%)
	Higher protective ability means more safe and comfortable	(n = 4, 10.5%)
	More expensive equipment is more comfortable	(n = 2, 5.2%)

## Data Availability

The datasets generated during and/or analyzed during the current study are available from the corresponding author upon reasonable request.
